# Antibody Responses Against *Anopheles darlingi* Immunogenic Peptides in *Plasmodium* Infected Humans

**DOI:** 10.3389/fcimb.2020.00455

**Published:** 2020-08-31

**Authors:** Berlin Londono-Renteria, Jehidys Montiel, Eric Calvo, Alberto Tobón-Castaño, Hugo O. Valdivia, Karin Escobedo-Vargas, Luz Romero, Maria Bosantes, Michael L. Fisher, Michael J. Conway, Gissella M. Vásquez, Audrey E. Lenhart

**Affiliations:** ^1^Vector Biology Laboratory, Department of Entomology, Kansas State University, Manhattan, KS, United States; ^2^Laboratory of Malaria and Vector Research, National Institute of Allergies and Infectious Diseases (NIAID/NIH), Rockville, MD, United States; ^3^Malaria Group, Universidad de Antioquia, Medellín, Colombia; ^4^U.S. Naval Medical Research Unit No. 6 (NAMRU-6), Callao, Peru; ^5^Asociación Benéfica PRISMA, Lima, Peru; ^6^Central Michigan University College of Medicine, Mount Pleasant, MI, United States; ^7^Division of Parasitic Diseases and Malaria, Entomology Branch, Centers for Disease Control and Prevention, Atlanta, GA, United States

**Keywords:** *An. darlingi*, peptides, antibodies, malaria, Colombia

## Abstract

**Introduction:** Malaria is still an important vector-borne disease in the New World tropics. Despite the recent decline in malaria due to *Plasmodium falciparum* infection in Africa, a rise in *Plasmodium* infections has been detected in several low malaria transmission areas in Latin America. One of the main obstacles in the battle against malaria is the lack of innovative tools to assess malaria transmission risk, and the behavioral plasticity of one of the main malaria vectors in Latin America, *Anopheles darlingi*.

**Methods:** We used human IgG antibodies against mosquito salivary gland proteins as a measure of disease risk. Whole salivary gland antigen (SGA) from *Anopheles darlingi* mosquitoes was used as antigen in Western blot experiments, in which a ~65 kDa protein was visualized as the main immunogenic band and sent for sequencing by mass spectrometry. Apyrase and peroxidase peptides were designed and used as antigens in an ELISA-based test to measure human IgG antibody responses in people with different clinical presentations of malaria.

**Results:** Liquid chromatography–mass spectrometry revealed 17 proteins contained in the ~65 kDa band, with an apyrase and a peroxidase as the two most abundant proteins. Detection of IgG antibodies against salivary antigens by ELISA revealed a significant higher antibody levels in people with malaria infection when compared to uninfected volunteers using the AnDar_Apy1 and AnDar_Apy2 peptides. We also detected a significant positive correlation between the anti-peptides IgG levels and antibodies against the *Plasmodium vivax* and *P. falciparum* antigens PvMSP1 and PfMSP1. Odd ratios suggest that people with higher IgG antibodies against the apyrase peptides were up to five times more likely to have a malaria infection.

**Conclusion:** Antibodies against salivary peptides from *An. darlingi* salivary gland proteins may be used as biomarkers for malaria risk.

## Introduction

Malaria is one of the most important parasitic diseases of the tropics and subtropics. Despite global decreases in malaria cases, Latin America has experienced an increase in cases since 2015. Colombia is among the top five Latin American countries with the highest numbers of malaria cases reported annually (Chaparro-Narvaez et al., 2016). In 2019 the number of malaria cases officially reported was 78,513 with Chocó, Nariño, Cordoba, and Antioquia, the departments with the highest number of malaria cases (31.4, 20.4, 11.6, and 9.3%, respectively) (Instituto Nacional de Salud, 2019). Several factors contribute to this phenomenon. First, more than 80% of the Colombian territory is suitable for the transmission of malaria due to the presence of a diverse number of mosquito species capable of serving as vectors of *Plasmodium* spp. (Rodriguez et al., 2011). Second, at least three species of *Plasmodium* cause infection in Colombia, with *Plasmodium vivax* as the main species, followed by *Plasmodium falciparum* and *Plasmodium malariae* (Arevalo-Herrera et al., 2012). Forced migration and precarious socioeconomic conditions also favor the spread and prevalence of *Plasmodium* spp. (Martens and Hall, 2000; Jitthai, 2013; Rodrigues et al., 2018).

Globally, most efforts to prevent malaria transmission focus on *P. falciparum*, the deadliest malaria parasite species. However, *P. vivax* is also a significant cause of morbidity and mortality (Chaparro-Narvaez et al., 2016; Geleta and Ketema, 2016; Zain Ul et al., 2016), and improved methods of monitoring its transmission are needed. Malaria transmission in Colombia occurs year-round, with peaks typically occurring between February and June (Ruiz et al., 2006). Malaria incidence is highly associated with changes in climatic conditions (i.e., temperature, elevation, and humidity) (Ruiz et al., 2006; Cassab et al., 2011). More importantly, ~87% of the reported cases come from people living in rural areas with limited access to health care (Rodriguez et al., 2011). Frequently, research about malaria transmission in these areas is restricted due to the lack of infrastructure to support research, including limited access to electricity.

Currently, the gold standard to determine malaria transmission intensity is the Entomological Inoculation Rate (EIR), which represents the number of infectious mosquito bites a person receives per night. This provides an indirect estimation of the risk of acquiring the disease. One of the challenges of this technique is the need to detect parasites in mosquitoes that are collected in the field. Mosquito trapping systems can be biased and overrepresent the most abundant species in a specific area. In addition, current methods are unable to pinpoint the exact individuals who have been bitten or whether there are differences in the number of bites different people receive. Thus, current surveillance efforts lack individualized, deployable, and cost-effective tools to measure human-vector contact and improve measurement of malaria transmission risk. Such tools would be particularly valuable in assessing the efficacy of vector control interventions in protecting people from malaria vector bites.

Our previous studies demonstrated that IgG antibodies against salivary proteins of major disease vectors correlate with the clinical presentation of dengue fever and malaria (Londono-Renteria et al., 2010; Londoño-Rentería et al., 2015a; Montiel et al., 2020a), and can be used to evaluate vector control interventions (Londono-Renteria et al., 2015c). In fact, IgG antibodies against a salivary peptide (gSG6-P1) from a major African malaria vector has been validated as a reliable marker to measure exposure to several *Anopheles* spp. (Poinsignon et al., 2009; Badu et al., 2012; Sagna et al., 2013). However, recent studies suggest that the gSG6 gene is not present in the subgenus *Nyssorhynchus*, which is the most prevalent American *Anopheles* subgenus (Arca et al., 2017). Although, previous studies have shown that the gSG6-P1 may be useful in areas of the New World where species belonging to subgenus other than *Nyssorhynchus* (Londono-Renteria et al., 2015b; Montiel et al., 2020a), there is a need to detect and validate novel biomarkers for exposure to these *Anopheles* spp. There are at least 40 *Anopheles* species in Colombia (Montoya-Lerma et al., 2011). The main vectors in the country are *Anopheles nuneztovari, Anopheles albimanus* and *Anopheles darlingi*—all three species within the subgenus *Nyssorhynchus* (Montoya-Lerma et al., 2011). Recently, we described several peptides that could be used to determine exposure to *Anopheles albimanus* bites, with antibodies against transferrin and salivary peroxidase proteins associated with the clinical presentation of malaria (Montiel et al., 2020a).

In this study, we evaluated the immunogenicity of salivary proteins in *An. darlingi* salivary gland antigen (SGA) and designed four peptides to measure IgG responses in people living in malaria endemic areas. The objective was to evaluate whether IgG antibodies against these peptides could be reliable markers to detect the risk of disease. *An. darlingi* is one of the most prolific mosquito vectors of malaria in Central and South America (Mirabello and Conn, 2006; Villarreal-Trevino et al., 2015; Prado et al., 2019), known for its behavioral plasticity and highly anthropophilic behavior. This mosquito species bite indoors and outdoors (endophagic/exophagic) and display exophilic/endophilic opportunistic behaviors (Moutinho et al., 2011; Reinbold-Wasson et al., 2012; Moreno et al., 2015). Our study suggests that IgG antibodies against *An. darlingi* salivary proteins are useful to assess malaria risk in areas where this species is endemic. This is the first study describing *An. darlingi* salivary peptides as potential tools to evaluate immune responses against mosquito saliva with the aim to improve accurate estimation of malaria transmission intensity, which is imperative for directing control efforts and predicting the effects of such interventions.

## Materials and Methods

### Ethical Considerations and Human Sample Collection

Serum samples from 179 participants were collected by active and passive case detection from November 2016 to October 2017 in Turbo and El Bagre as part of a malaria study in Colombia. These represent two malaria endemic areas with differing malaria incidence as measured by the Annual Parasite Index (API: confirmed cases during 1 year/population under surveillance ×1,000): the API in Turbo was 1.22 in 2016 and 0.77 in 2017, while malaria incidence in El Bagre was higher, with an API of 24.7 in 2016 and 21.3 in 2017 (Londoño-Rentería et al., 2015a; Londono-Renteria et al., 2020; Montiel et al., 2020b). Participant characteristics are described in Table 1. No concurrent entomological data was collected for this study. However, these field sites were selected based on a previous study reporting entomological data, where more than 96% of mosquitoes collected in Turbo were *An. albimanus*, while more than 70% of the mosquitos in El Bagre were *An. darlingi* (Gutierrez et al., 2009). Human sample collection and testing protocols were approved by the IRB of Kansas State University (IRB #8956 and IBC #1206) and the Medical School at the Universidad de Antioquia in Medellín, Colombia (Record 011 dated 28 July 2016).

**Table 1:** Description of the serum samples by malaria infection status and location.

**Malaria infection status**	**Turbo**	**El bagre**	**Total**
*P. falciparum*	8	18	26
*P. vivax*	36	29	65
Uninfected	45	43	88
Total	89	90	179

### Detection of Malaria Parasites

On-site malaria diagnosis was done by microscopy, then confirmed by nested PCR (nPCR) at the Universidad de Antioquia (Colombia) following methods described previously (Londoño-Rentería et al., 2015a; Londono-Renteria et al., 2020). Based on these results, samples were classified as malaria positive or malaria negative, based on the presence/absence of parasites or a positive PCR result. In case of a disagreement, the PCR results was taken as the final diagnosis.

### *An. darlingi* Mosquito Rearing

All *An. darlingi* were reared in the insectary at the Naval Medical Research Unit No. 6 (NAMRU-6) in Iquitos, Peru as described previously (Villarreal-Trevino et al., 2015). Larvae were reared in a dedicated larvae room (26.8 ± 0.7°C and 76.1 ± 6.3% relative humidity, and 12-h light:12-h dark photoperiod) and adults were maintained in a dedicated adult insectary room (25.9 ± 0.8°C and 69.7 ± 5.7% relative humidity, and 12-h light:12-h dark photoperiod) (Villarreal-Trevino et al., 2015). Eight to ten- day old adult mosquitoes were used for dissections of salivary glands. These mosquitoes were blood fed with chicken blood at an age of 3–5 days post-emergence, and provided with 10% sucrose solution *ad libitum*.

### Salivary Gland Antigen (SGA) Preparation

Female *An. darlingi* mosquitoes were cold-anesthetized, washed in 70% ethanol, and placed in PBS (pH 7.2) for salivary gland dissection. Salivary glands were pooled (100 mosquitoes per pool for a total of two pools) and placed in 1X PBS. To prepare the antigen extract, the pool of salivary glands was allowed to freeze at −80°C and thaw at 4°C four times to induce cell rupture and release of proteins; the resulting SGA was kept in PBS at −80°C until use. Protein concentration was determined using a Thermo Scientific NanoDrop™ (Thermo Fisher Scientific, Wilmington, DE, USA) (Londono-Renteria et al., 2013, 2020; Montiel et al., 2020a).

### Immunoblotting

To identify immunogenic proteins in *An. darlingi* SGA, pooled sera from ten uninfected subjects were used (five subjects from Turbo and five subjects from El Bagre) were used in the Immunoblotting experiments performed according to Londono-Renteria et al. (2010) (Londono-Renteria et al., 2010). In brief, 2 μg/well of SGA was separated in a 4–15% polyacrylamide gel (BioRad) at 100 V and transferred to a nitrocellulose membrane (Turbo PVDF, 0.45-m pore size, 8.5 by 13.5 cm, Biorad) in a Transblot Turbo following the standard 30 min transference protocol. Membranes were blocked for 1 h with blocking buffer (2% milk in PBST) and incubated with pooled human sera (1/250) at 4°C overnight. Each membrane was washed five times with wash solution (1X PBST) and incubated with HRP-conjugate anti human-IgG, in a 1/1,000 dilution during 1 h at 37°C. Color development was obtained with HRP chromogenic substrate TMB (Novex, Invitrogen). A pre-stained molecular weight marker of 16.5Ð*210 kDa (BioRad) was used for the estimation of the size of the proteins.

### Liquid Chromatography/Mass Spectroscopy (LC/MS)

A band of 65 kDa was identified by human serum in the immunoblot. Gel bands corresponding to the immunogenic bands were excised and sent in duplicate to the National Institutes of Health (NIH/NIAID) for identification by mass spectrometry (MS). Briefly, the samples were reduced in solution containing 50 mM HEPES (pH 8.0) 10% acetonitrile and 5 mM DTT for 40 min at 37°C. After cooling to room temperature, the samples were mixed at 15 mM with iodoacetamide. After 15 min of alkylation, 200 ng of trypsin was added and the samples were incubated at 37°C for 15 h in a final volume of 40μl. The solution was evaporated to near dryness under a vacuum at 50°C. Twenty-five microliters of 0.1% trifluoroacetic acid was added and the pH was adjusted to 2.5 with the addition of 10% trifluoroacetic acid. Samples with an estimated protein content of <2 μg were desalted and concentrated with C18 μ Zip Tips. Samples containing up to 10μg were desalted with C18 OMIX 10 solid phase extraction tips. The digests were eluted with 0.1% TFA, 50% acetonitrile and dried under vacuum. The peptides were dissolved in 12 μl 0.1% formic acid, 3% acetonitrile which was used as the injection solvent. Digested peptides were subjected to the LC/MS analysis using an Orbitrap Fusion mass spectrometer (ThermoFisher Scientific, West Palm Beach, FL) connected with EASY nLC 1,000 liquid chromatography (LC) system. Nano-LC was carried out with a 5 μL injection onto a PepMap 100 C18 3-μm trap column (2 cm, ID 75 μm) and a 2 μm PepMap RSLC C18 column (25 cm, ID 75 μm), both from ThermoFisher Scientific. The LC was operated at a 300 μL/min flow rate with a 100-min linear gradient from 100% solvent A (0.1% formic acid, and 99.9% water) to 40% solvent B (0.1% formic acid, 20% water, and 79.9% acetonitrile) followed by a column wash. A standard data-dependent acquisition performed with a full MS spectrum was obtained by the Orbitrap for m/z 400–2,000 at the resolution of 120,000 with EASY-IC calibration. The precursor ions, with charges from two to eight, were selected, isolated (1.6 m/z window), fragmented by CID, then scanned by the Ion Trap. Survey scans were performed every 2 s and the dynamic exclusion was enabled for 30 s.

Acquisitions were searched against the NCBI-nr (05/2019) proteome and the cRAP.fasta database (theGPM.org) using PEAKS v10 (Bioinformatics Solutions Inc, Ontario, Canada) using a semi-tryptic search strategy with tolerances of 6 ppm for MS and 0.5 Da for MS/MS and carbamidomethylation of cysteine as a fixed modification and oxidation of methionine as a dynamic modification allowing for two missed cleavages. Peptides were filtered with a 0.5% FDR using a decoy database approach and a two spectral matches/peptide requirement.

### *Anopheles* Immunogenic Peptide Selection and *Plasmodium* Antigens

Immunogenic peptides were designed from the most abundant identified proteins: apyrase (Protein Accession # ETN63669.1) and peroxidase (Protein Accession # ETN66035.1) (Table 2). Proteins were analyzed for the presence of signal peptide (signal P) sequence using the SignalP 5.0 server and for sequence homologies (at least 50% identity with *E*-value 1 × 10–5) to *Anopheles* and other major culicid disease vector species using online BLAST program. Protein sequence and structure analyses were then performed for the top *Anopheles*-specific proteins using the Protean 3D package of the DNASTAR software (DNASTAR Inc., Madison, WI, USA) (Londono-Renteria et al., 2020). The analysis of linear B-cell epitopes, antigenic regions, flexible regions (turns), hydrophobic regions, stability and/or charge density were conducted by importing the FASTA files from NCBI of sequences of interest into SVMTriP (Center for Plant Innovation, University of Nebraska, Lincoln, NE, USA). Highly antigenic, stable, flexible, charged and <50% hydrophobic regions that were specific to *Anopheles* mosquitoes (>50% homology) were selected using the software for 18–22 amino acid peptides (Table 3). The AnDar_PeroX1, AnDar_PeroX2, AnDar_Apy1, and the AnDar_Apy2 peptides of interest were sent for synthesis to GenScript (Piscataway, NJ, USA).

**Table 2:** List of proteins identified in an ~65 kDa protein band from the western-blot using a pool of serum from study participants.

**Protein accessions**	**Description**	**Avg. mass**
ETN63669.1	Apyrase (*An. darlingi*)	63,178
ETN66035.1	Oxidase/peroxidase (*An. darlingi*)	65,566
ACI30180.1	Putative salivary protein SG1B (*An. darlingi*)	51,295
ETN62038.1	ATP synthase beta subunit (*An. darlingi*)	53,768
ETN61268.1	Protein disulfide isomerase (*An. darlingi*)	54,542
ETN64733.1	Venom allergen 5 (*An. darlingi*)	29,387
ETN59462.1	ATP synthase alpha subunit mitochondrial (*An. darlingi*)	59,383
ACI30121.1	SG1-like salivary protein (*An. darlingi*)	44,890
ETN65121.1	Pyruvate kinase (*An. darlingi*)	57,551
ACI30100.1	30 kDa salivary antigen family protein (*An. darlingi*)	25,628
ETN58076.1	Deoxyribonuclease I (*An. darlingi*)	45,530
ETN64604.1	Malic enzyme (*An. darlingi*)	69,409
ETN63240.1	Protein disulfide isomerase (*An. darlingi*)	55,374
ETN62075.1	Apyrase (*An. darlingi*)	62,276
ETN61375.1	Catalase (*An. darlingi*)	62,481
ETN57775.1	Actin (*An. darlingi*)	31,545
ACI30046.1	Long form D7 salivary protein (*An. darlingi*)	37,105

**Table 3:** *An. darlingi* peptide sequences from the apyrase and the peroxidase/oxidase proteins identified by mass spectrometry.

**Peptide name**	**Peptide sequence**	**a.a. position**
AnDar_PeroX1	RGQCDSTSPYRTYDGRCNNLQN	19–40
AnDar_PeroX2	GQCDSTSPYRTYDGRCNNLQNP	20–41
AnDar_Apy1	GGHSHSFLFSPDSDQPYNKQDT	249–270
AnDar_Apy2	HMNDLHARFDETSNKSSKCRSD	39–60

Two recombinant proteins representing the *Plasmodium* Merozoite Surface Protein from *P. falciparum* (Pf-MSP) (Montiel et al., 2020a) and *P. vivax* (Pv-MSP) (Fitzgerald, USA) were used to evaluate antibody responses against the parasite.

### ELISA Testing

ELISA conditions were standardized as published elsewhere (Londono-Renteria et al., 2010) and the test was used to measure IgG antibodies against total SGA and individual peptides in human samples. Briefly, 96-well ELISA plates (UltraCruz® ELISA Plate, Santa Cruz Biotechnology, Dallas, TX) were coated with 100 μL/well of 0.5 μg/ml of *An. darlingi* SGA or 2 μg/ml of each peptide in 1X PBS and incubated overnight at 4°C. Plates were blocked for 1 h at room temperature with 2% milk in 1X PBST (blocking buffer) and incubated with 100 μL/well of a 1/100 serum dilution in blocking buffer at 37°C for 2 h. Plates were washed three times with wash solution (1 × PBS and 0.1% Tween) and incubated with 100 μL/well of horseradish peroxidase (HRP)-conjugated goat anti-human IgG (1:1,000), antibodies (Abcam, Cambridge, UK) at 37°C for 1.5 h. Colorimetric development was carried out using tetra-methyl-benzidine (Abcam) as a substrate and incubated for 2 min before the reaction was stopped using 2 N sulfuric acid. Absorbance was measured at 450 nm. Each sample was tested in duplicate. Three controls were included in each plate: (1) control blank: two wells without SGA to control for non-specific induction of color for any of the reagents used in the test; (2) negative control: two wells with SGA but without human serum to control for any non-specific color induction of the coating antigen; and (3) positive control to control for plate to plate variation and normalize OD values. To determine a cut off value of exposure to *Plasmodium* antigens and *An. darlingi*, we tested serum from 12 volunteers living in the US with no history of travel to South America. The OD cut off values to establish exposure were set at 0.167 for Pf-MSP1, 0.153 for Pv-MSP1 and 0.396 for *An. darlingi* SGA.

### Data Analysis

After testing for normality, we concluded that the data did not meet the normality requirement. Consequently, we performed non-parametrical statistical tests. Specifically, we used the Mann-Whitney *U*-test to compare two independent groups (i.e., malaria positive vs. negative) and the Kruskal-Wallis test to compare more than two independent groups (i.e., negative, *P. vivax* malaria and *P. falciparum* malaria). Spearman Correlation Coefficients were calculated to measure the strength of association between IgG antibodies against salivary proteins and IgG antibodies against *Plasmodium* antigens as well as age. Odd ratios and Fisher's exact test were used to assess significance of risk calculation. OD values were categorized as low (less than the median OD) or high (equal or above the median OD) for each peptide: AnDar_PeroX1 (median OD = 0.579), AnDar_PeroX2 (median OD = 0.6612), AnDar_Apy1 (median OD = 0.4410) and the AnDar_Apy2 (median OD = 0.4286). All tests were considered significant with a *p-value* < 0.05.

## Results

### Immunogenic Proteins in *An. darlingi* SGA

**Figure 1:**
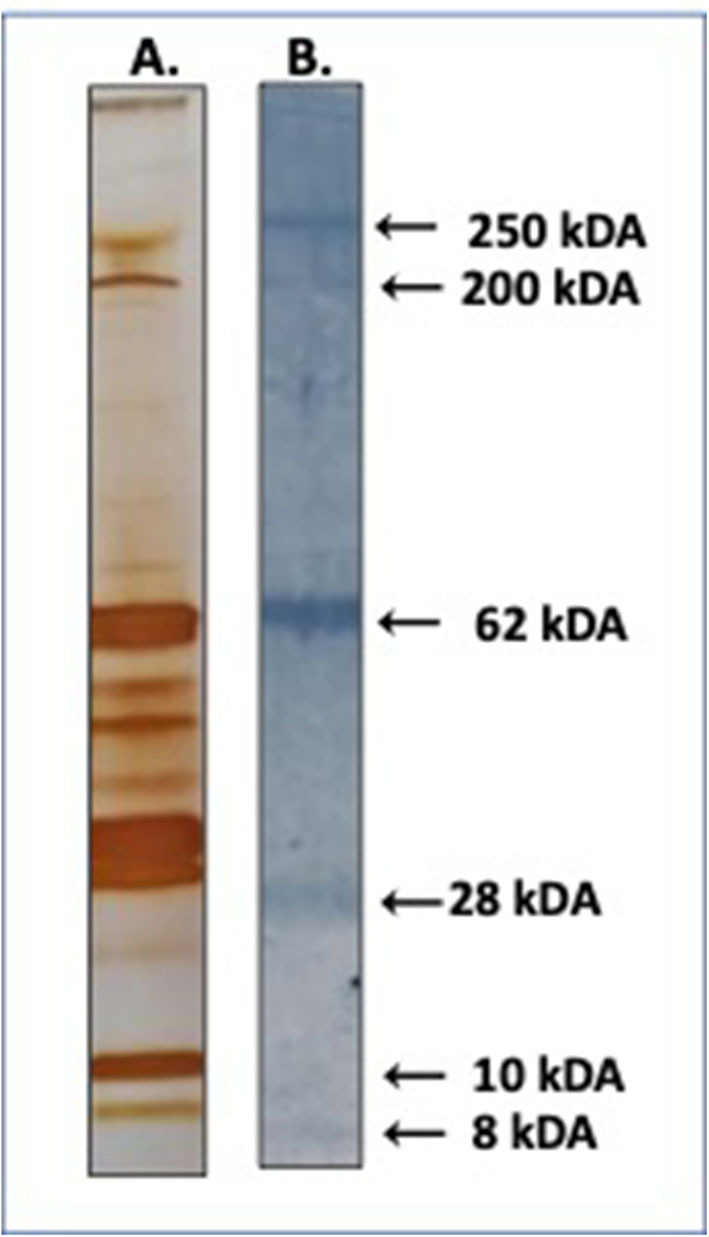
Immunoblot of *An. darlingi* SGA. Pooled serum from uninfected individuals exposed to *An. darlingi* bites was used to identify immunogenic proteins. **(A)** Silver stain of whole SGA. **(B)** Immunoblot.

Western blot testing revealed four major immunogenic bands (~250, ~62, ~28, and ~8 kDa) (Figure 1). The most prominent band was a 65 kDa band that was excised and sent for sequencing. Seventeen proteins were identified by LC-MS (Table 2). The most abundant proteins found were a ~63 kDa apyrase (Accession# ETN63669.1) and a 65 kDa oxidase/peroxidase (Accession# ETN66035.1). Two peptides were designed from each of these proteins (AnDar_Apy1, AnDar_Apy2, AnDar_PeroX1, and AnDar_PeroX2) to evaluate immune responses to these specific salivary proteins in people with active malaria infections. The specific sequence of each peptide is described in Table 3. Apyrases as well as salivary peroxidases are ubiquitous proteins. The *An. darlingi* apyrase described in the current study has a 63% identity with an apyrase of *Anopheles gambiae* (AGAP011971), and 49% with an apyrase from *Aedes aegypti* (AAEL006347) and an apyrase of *Cx. quinquefasciatus* (CPIJ011010). No significant similarity was found with *An. albimanus* apyrases. The *An. darlingi* salivary oxidase/peroxidase has a 87.8% identity with the *An. albimanus* peroxidase (AAD22196.1). The *An. darlingi* oxidase/peroxidase also presents a 53% similarity with *An. gambiae* (AGAP010735) while similarity with *Ae. aegypti* (AAEL000507) and *Cx. quinquefasciatus* (CPIJ017579) was 48%. However, the selected immunogenic peptides align with identities between 56 and 85% with these proteins (Supplementary Table 1).

### Antibody Responses Against Salivary Proteins

**Figure 2:**
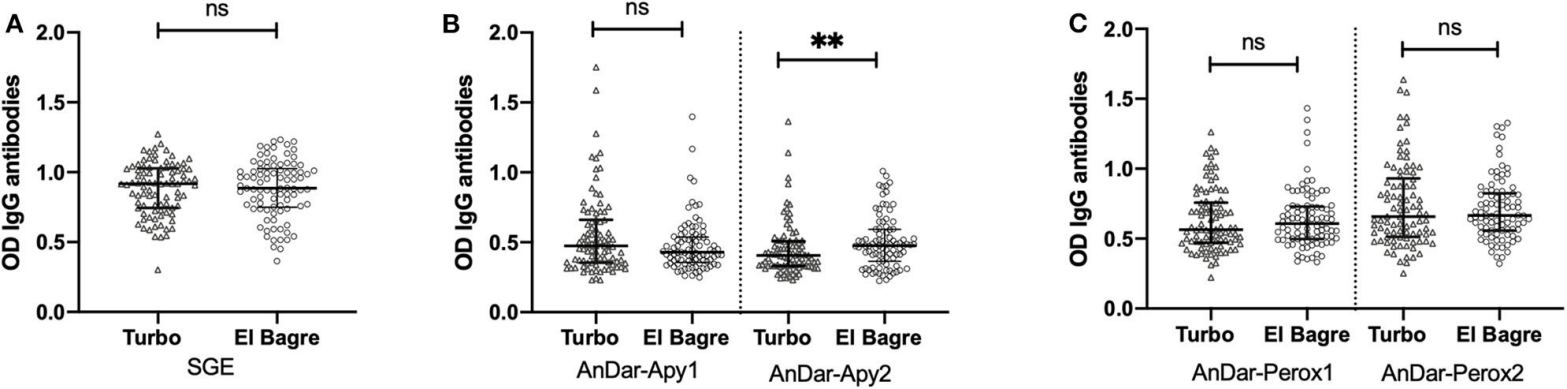
Comparison of IgG antibody levels against *An. darlingi* SGA **(A)** and peptides from Apyrase **(B)** and Peroxidase **(C)** between the study sites (Turbo and El Bagre). ns denotes not significant, *p* < 0.05 (* = 0.01 to 0.05, ** = 0.001 to 0.01, and *** = 0.0001 to 0.001).

**Figure 3:**
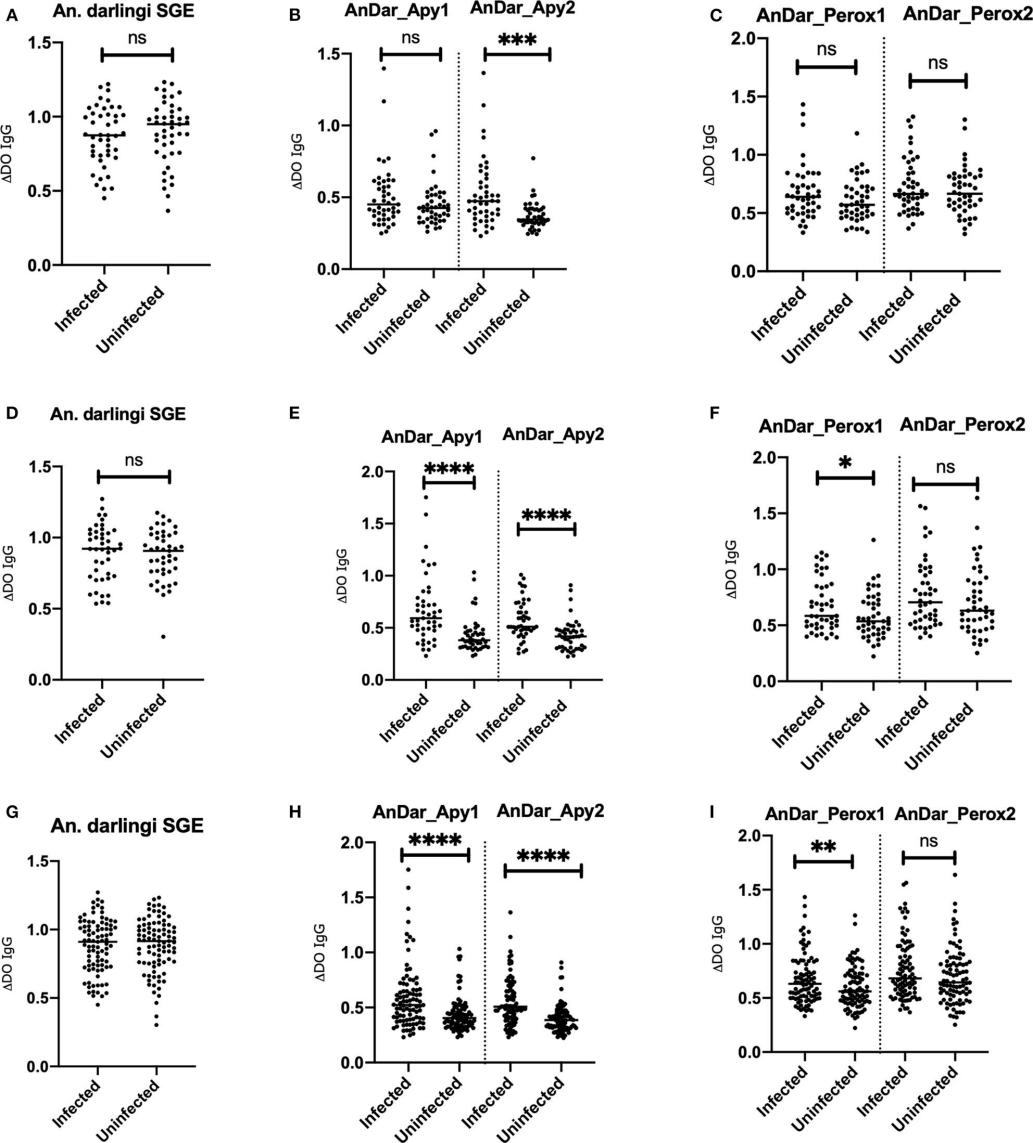
Comparison of IgG antibody levels against *An. darlingi* salivary antigens in samples with malaria infections and uninfected samples from Turbo **(A–C)**, El Bagre **(D–F)** and both sites combined **(G–I)**. ns denotes not significant, *p* < 0.05 (* = 0.01 to 0.05, ** = 0.001 to 0.01, and *** = 0.0001 to 0.001).

Serum from a total of 107 females and 72 males with ages between 2 and 83 years old (Age average = 25.1 years old) were included in this study. No significant differences were detected when comparing IgG antibody levels against any of the antigens from either Turbo or El Bagre samples with the exception of AnDarApy2 which was significantly higher in El Bagre (Mann-Whitney test *p* = 0.0097) (Figure 2). When comparing IgG antibody levels among groups by their infection status (malaria negative vs. malaria positive), we did not detect significant differences between the groups from either field site using the whole SGA as antigen (Mann-Whitney test *p* > 0.05) (Figure 3). However, when using the apyrase peptides, we found that antibodies against AnDar-Apy2 were significantly higher in malaria positive patients in both Turbo (Mann-Whitney test, *p* = 0.0001) and El Bagre (Mann-Whitney test, *p* < 0.0001), while AnDar-Apy1 was higher in malaria positive samples only in El Bagre (Mann-Whitney test, *p* < 0.0001). Interestingly, the peroxidase-derived peptides, only showed significant differences between infected and uninfected samples from El Bagre, where AnDar-PeroX1 was significantly higher in malaria infected people (Mann-Whitney test, *p* = 0.0497). When comparing the antibody levels regardless of the field site, both of the apyrase peptides and AnDarPeroX1 were significantly higher in malaria infected samples.

### Antibody Responses Against *Plasmodium* and Mosquito Salivary Proteins

**Figure 4:**
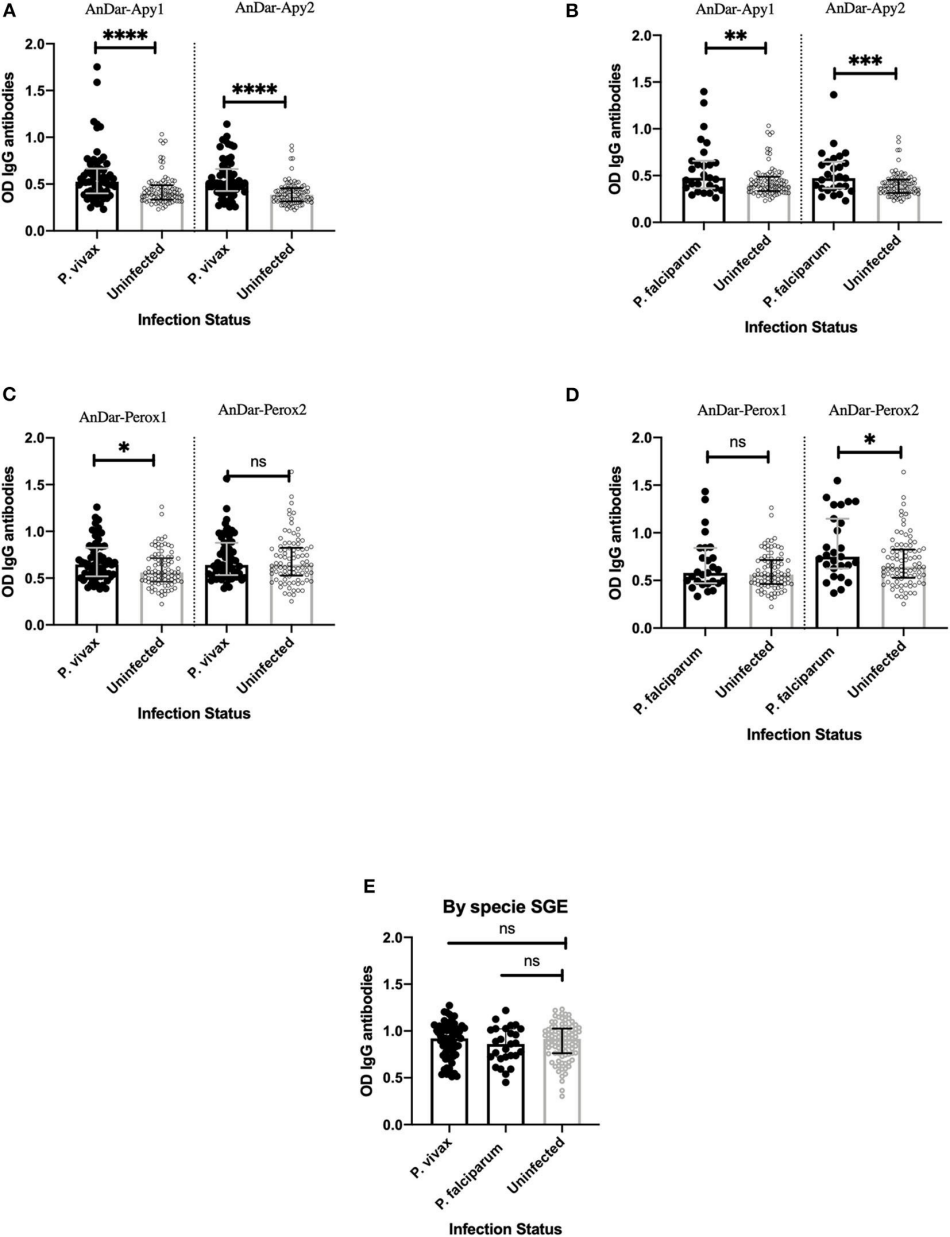
Comparison of IgG antibody levels against *An. darlingi* apyrase peptides **(A,B)**, peroxidase peptides **(C,D)**, and SGA **(E)**, in samples from individuals infected with either *P. falciparum* or *P. vivax*. ns denotes not significant, *p* < 0.05 (* = 0.01 to 0.05, ** = 0.001 to 0.01, and *** = 0.0001 to 0.001).

Spearman correlation analysis showed significant associations between three of the salivary proteins (AnDar_Apy1, AnDar_Apy2, and AnDar_PeroX2) and either PvMSP1 or PfMSP1 (Spearman rank correlation test, *p* < 0.05). When divided by malaria infection status, a significant positive association between IgG antibodies against AnDarApy2 and AnDarPeroX2 was maintained with both PvMSP1 and PfMSP1 in both malaria infected and uninfected individuals (Table 4). No association with either *Plasmodium* antigen was detected when comparing IgG antibodies against AnDarPeroX1. When comparing whether there were significant differences in the IgG antibody levels against any salivary antigens and the species of *Plasmodium* causing infection, we found that antibodies against both apyrases peptides were significantly higher in people with *P. vivax* infection (Mann-Whitney test, *p* < 0.0001) and *P. falciparum* [Mann-Whitney test, *p* = 0.0011 (AnDar_Apy1), *p* = 0.0005 (AnDar_Apy2)] when compared against IgG antibodies in uninfected participants. In the case of IgG antibodies against the peroxidase peptides, we found only significant higher antibodies against AnDar_Perox1 in *P. vivax* infection (Mann-Whitney test, *p* = 0.0114 (*P. vivax*), *p* = 0.1285 (*P. falciparum*), while antibodies against AnDar_Perox2 were significantly higher only when comparing *P. falciparum* infected against uninfected (Mann-Whitney test, *p* = 0.6464 (*P. vivax*), *p* = 0.0131 (*P. falciparum*). No differences were found with SGE antibodies (Mann-Whitney test, *p* > 0.05) (Figure 4).

**Table 4:** Correlation analysis between IgG antibodies against *An. darlingi* salivary antigens and IgG antibodies against *P. falciparum, P. vivax* showing samples grouped by infection status.

**All samples (*n* = 179)**	**PvMSP1 (*p*-value)**	**PfMSP1 (*p*-value)**
AnDar_Apy1	0.16 (0.0305)^*^	0.16 (0.0290)^*^
AnDar_Apy2	0.39 (0.0001)^†^	0.35 (0.0001)^†^
AnDar_PeroX1	0.15 (0.0503)^*^	0.12 (0.1208)
AnDar_PeroX2	0.37 (0.0001)^†^	0.26 (0.0004)^†^
SGE	−0.06 (0.4245)	−0.03 (0.6104)
**Malaria infected (*****n*** **=** **89)**	**PvMSP1 (*****p*****-value)**	**PfMSP1 (*****p*****-value)**
AnDar_Apy1	0.06 (0.5544)	0.15 (0.1557)
AnDar_Apy2	0.29 (0.0045)^*^	0.32 (0.0018)^*^
AnDar_PeroX1	0.13 (0.1971)	0.19 (0.0741)
AnDar_PeroX2	0.35 (0.0008)^†^	0.26 (0.0106)^*^
SGE	−0.04 (0.6879)	0.00 (0.9975)
**Malaria uninfected (*****n*** **=** **90)**	**PvMSP1 (*****p*****-value)**	**PfMSP1 (*****p*****-value)**
AnDar_Apy1	0.15 (0.1689)	0.11 (0.2770)
AnDar_Apy2	0.41 (0.0001)^†^	0.33 (0.0013)^†^
AnDar_PeroX1	0.09 (0.3544)	−0.00 (0.9881)
AnDar_PeroX2	0.39 (0.0001)^†^	0.24 (0.0184)^*^
SGE	−0.06 (0.5791)	−0.06 (0.5444)

* p < 0.05;

† *p < 0.001*.

### Malaria Risk and IgG Antibodies Against *An. darlingi* Salivary Proteins

Odd ratios were calculated to determine the risk of malaria infection based on the levels of IgG antibodies against whole SGA and the individual salivary peptides. Our analysis indicated that samples with high levels of antibodies against apyrase peptides had 3.1 (AnDar_Apy1) and 5.1 (AnDar_Apy2) times higher probability of being positive for malaria than samples with lower antibody levels (Fisher's exact test, *p* < 0.0001). Significant odd ratios were not observed when comparing IgG levels of SGA, AnDar_Perox1 or AnDarPerox2 (Table 5).

**Table 5:** Odds ratio calculations to measure risk of malaria infection based on level of IgG antibodies against *An. darlingi* salivary proteins.

**Salivary antigen**	**Odds ratio**	**95% Confidence interval**	***P*-value**
AnDar_Apy1	3.1	1.63197–6.025945	**0.0001**
AnDar_Apy2	5.1	2.617258–10.25419	**0.0001**
AnDar_PeroX1	1.8	0.9509861–3.372674	0.0526
AnDar_PeroX2	1.4	0.7292346–2.560444	0.2967
SGE	0.9	0.5114593–1.788728	0.8815

*Bold values indicates p > 0.05*.

## Discussion

*An. darlingi* is one of the most important malaria vectors in the Americas, and is associated with transmission of both *P. falciparum* and *P. vivax* (de Arruda et al., 1986; Flores-Mendoza et al., 2004). Some data suggest that infected *An. darlingi* tend to bite more outdoors and in the early evening, which reduces the effectiveness of bed nets (Moreno et al., 2015; Newell et al., 2018). The (EIR) is the current gold standard to estimate malaria transmission intensity. However, this technique is labor intensive and requires sensitive methods to accurately detect *Plasmodium* infection in field-captured mosquitoes. In addition, it is impossible to track individual human exposure to infective bites and estimation of risk that can be later used to determine local transmission dynamics. Tools capable of monitoring human-vector contact are needed to more accurately estimate disease risk. Here, we evaluated the IgG responses against *An. darlingi* salivary proteins and showed through ELISA that peptides against a salivary apyrase and peroxidase are suitable antigens to estimate the risk of suffering a malaria infection.

In an effort to identify candidate salivary peptides from *An. darlingi* salivary proteins, an immunogenic ~65 kDa protein band was identified by antibodies in serum collected from study participants. LC-MS revealed 17 salivary proteins, with apyrase and a peroxidase as the most predominant proteins. Most of the identified proteins have previously been associated with blood feeding. Salivary apyrases hydrolyze ATP and ADP inhibiting platelet aggregation. They are associated with host-seeking behavior and the ability of mosquitoes to find blood vessels (Calvo et al., 2006). Infection status with human pathogens influences mosquito behavior and changes the content of salivary gland and salivary secretions (Chisenhall et al., 2014a,b). A previous study suggested that apyrase activity decreases as sporozoite load increases, rendering the infected mosquitoes more likely to probe and re-feed after an infectious first blood meal (Thievent et al., 2019). Each probing event increases the likelihood of injecting sporozoites into the skin, which increases the chance of infection. Salivary proteins are also injected during each probing event (Ribeiro, 2000; Chisenhall et al., 2014a), which may explain the high antibody responses against salivary proteins in people with active malaria infection. Interestingly, immunization with mosquito saliva leads to the development of anti-apyrase antibodies, which can block its enzymatic activity. However, a decrease in feeding or probing time was not observed in immunized mice even in the presence of higher titers of anti-apyrase antibodies (Mathews et al., 1996). Previous studies have shown changes in salivary gland physiology of infected mosquitoes (Cotama et al., 2013; Pinheiro-Silva et al., 2015). It is unclear if infection with either *P. vivax* or *P. falciparum* changes the expression profile of apyrase or peroxidase proteins in the salivary gland of *An. darlingi* mosquitoes. This information would be helpful to determine a potential function of these proteins in malaria transmission by *An. darlingi* mosquitoes.

Besides the apyrase and the salivary peroxidase described above, other important salivary proteins that had been previously reported were also detected in this study. First, two members of the SG1 family were detected: the putative salivary protein SG-1B along with a SG1-like salivary protein. The SG-1B appears to be uniquely expressed in the female salivary glands, while the SG1-like can also be expressed in males (Arca et al., 2005). Recently, a member of the SG1 family, TRIO protein, was evaluated as a potential vaccine candidate against *Plasmodium* infection since immunization against these proteins induced partial protection against *Plasmodium berghei* infection. Protection was further improved when animals were co-immunized with saliva and parasite proteins (Dragovic et al., 2018). Second, a long form D7 protein was also detected. Our previous studies demonstrated the immunogenicity of this family of proteins in *Ae. aegypti* saliva (Londono-Renteria et al., 2018). The D7 protein family has been identified as a member of an odorant binding protein (OBP) superfamily known to bind biogenic amines such as histamine, playing an important role as mediators of inflammation and vascular permeability, and facilitating blood feeding (Calvo et al., 2006). Finally, a 30 kDa salivary antigen family protein was identified. These proteins are exclusively found in the salivary glands of adult female mosquitoes and previous work suggested that they may inhibit platelet aggregation (Yoshida and Watanabe, 2006; Calvo et al., 2007b). A venom allergen 5 was also identified, and this family of proteins is found ubiquitously in animals and plants. Their specific function in *Anopheles* mosquitoes is still unknown, but it is thought to function either in the suppression of the host immune system, or as an anticoagulant in a wide range of hematophagous arthropods (Ribeiro and Francischetti, 2003; Calvo et al., 2007a).

It is important to note that whole salivary gland proteins contained in the SGA have been successfully used as antigen to detect IgG antibodies in vertebrate serum (Waitayakul et al., 2006; Badu et al., 2012). However, salivary gland dissection is a tedious process requiring skilled personnel (Coleman et al., 2007) and a continuous source of mosquito salivary glands. Not all mosquitoes are easily colonizable, and although using salivary glands from field mosquitoes can offer a better approximation of the antigens that people are actually exposed to (Andrade et al., 2009), it can be challenging to collect them in sufficient numbers and maintain a proper cold chain and protein stabilization before they are processed for antigen preparation (Fontaine et al., 2011a). Thus, the use of recombinant proteins or peptides is advantageous, particularly when consistency and reproducibility are desired. In that regard, we selected four peptides from the two main immunogenic proteins found after sequencing of the ~65 kDa band, an apyrase and a salivary peroxidase. The ELISA results revealed that when using the AnDar_Apy1, AnDar_Apy2, and AnDar_PeroX1 peptides as antigens, the IgG antibody levels were significantly higher in samples with active malaria infection as compared to uninfected samples. These results are consistent with our recent study where we observed that malaria-infected samples had significantly higher antibody levels against *An. albimanus* salivary peptides (Londono-Renteria et al., 2020).

Previous studies associated this increase in antibodies with a potentially higher exposure to mosquito bites (Fontaine et al., 2011b). Moreover, this increase in IgG antibodies may also be the result of saliva-dependent activation of immune cells leading to the production of cytokines such as IL4 and IL10 (Vogt et al., 2018). IL4 is involved in the activation of B cells and their differentiation to plasma cells (Granato et al., 2014) and recent studies suggest that IL10 promotes IgG4 antibodies (Jeannin et al., 1998), which is the main antibody subtype that recognizes arthropod salivary antigens (Brummer-Korvenkontio et al., 1994; Cardenas et al., 2019). Further studies are needed to determine the mechanisms involved in the differential antibody profiles between people residing under the same conditions but with different malaria infection status.

In contrast to a previous study in Brazil (Andrade et al., 2009), we did not find significant differences between malaria-infected and uninfected samples when using SGA. One of our previous studies showed the possibility of discrete differences in the responses against *Anopheles* SGA from two different mosquito colony strains, with those differences potentially associated with differences in geographical origins as well as the time of colonization (Londono-Renteria et al., 2020; Montiel et al., 2020a). Another study detected important differences in salivary gland content from wild and colony maintained arthropods from the same species (Maldonado-Ruiz et al., 2019). Consequently, it is possible that the difference between this current study and the one performed in Brazil may reside in the source of the SGA and its components. Specifically, in this study, we used SGA from a recently colonized strain of *An. darlingi* in Iquitos, Peru, while the previous study used SGA from field-caught mosquitoes in Brazil (Andrade et al., 2009).

Regardless, analysis of the immunogenic proteins among mosquito strains may reveal shared immunogenic proteins that represent suitable candidates to measure risk of disease irrespective of the geographical area or the strains. Thus, it was important to identify the evolutionarily conserved regions within immunogenic proteins as we seek to develop widely applicable biomarkers of malaria risk. Only AnDar_Apy2 was significantly higher in El Bagre, the area with historically higher abundance of *An. darlingi* relative to Turbo (Gutierrez et al., 2009, 2010; Rosero et al., 2013); all other peptides did not show differences between the two sites. The lack of difference in antibody levels against most of the peptides between the two study sites may be explained by the 56 and 80% identity with other major salivary proteins representing a source of cross-reactivity. However, since we did not include concurrent mosquito collection data, we cannot confirm the concurrent *An. darlingi* population density at the time of this study. Thus, the results presented here are more sensitive at correlating antibodies against salivary proteins with presence of malaria infection rather than *An. darlingi* bite intensity.

Previous studies suggested that there is a significant association between the level of IgG antibodies against the most prominent *Plasmodium* antigens and the development of human immunity against malaria symptoms (Doolan et al., 2009; Rodriguez-Barraquer et al., 2018). Among these antigens, MSP-1 is considered as one of the more important in the development of immunity against malaria and has been investigated as a potential vaccine candidate. Immunity against malaria is thought to be acquired through chronic and sustained exposure to *Plasmodium* antigens (Rodriguez-Barraquer et al., 2018). However, recent evidence suggests that immune responses against salivary proteins may also contribute to the development of immunity against disease (Manning et al., 2018). Our current study revealed that the level of IgG antibodies against AnDar_Apy 2 and the AnDar_PeroX2 were significantly associated with IgG levels against both PvMSP1 and PfMSP1 in all study groups, suggesting a positive association between intensity of exposure to malaria antigens and both peroxidase and apyrase salivary proteins. Furthermore, when measuring the risk of malaria infection through odd ratio calculations, a significant increase in risk was only observed in antibodies against the apyrase peptides. Previous study showed an association between the level of antibodies against PfMSP1 and the only currently validated salivary biomarker from the *An. gambiae* salivary peptide, gSG6-P1 (Badu et al., 2012; Ya-Umphan et al., 2018). Our results suggest that AnDarApy1 and AnDarApy2 may be more suitable biomarkers to estimate malaria risk in areas where *An. darlingi* mosquitoes are endemic. Also interesting is that the correlation between *Plasmodium* antigens and the apyrase peptides salivary was also significant in the uninfected population. It is important to remember that infected individuals present significantly higher IgG antibodies. People in endemic areas may encounter a significant number of bites containing infective sporozoites throughout their lives. Furthermore, in endemic areas for *P. vivax*, as the one where this study was carried out, is very difficult to differentiate a recent infection from a recrudescence, and people may suffer several malaria infections in a year. Since this study did not included follow up of patients, we cannot pinpoint the time of the most recent infection. However, our study showed a significant higher antibody levels against both apyrases peptides and the AnDar_Perox1 in *P. vivax* infected populations suggesting the relevance of these peptides to measure risk of infection with the most prevalent species circulating in this area. Further studies are urgently needed to determine the persistence of antibodies against the salivary proteins and correlate with previous/recent malaria episodes.

Although further studies including a greater number of serum samples from different geographical areas are needed to validate these peptides as biomarkers of both malaria risk and human-vector contact, the current study represents an important advance in evaluating immune responses against mosquito salivary peptides in Latin America.

## Conclusion

Peptides derived from the *An. darlingi* salivary apyrase and peroxidases are suitable candidate markers for measuring human IgG antibody responses that may be associated with the risk of malaria infections.

## Data Availability Statement

All datasets generated for this study in the sequencing data are included in the article and the Supplementary Material. Other raw data will be available upon request sent to blondono@ksu.edu.

## Ethics Statement

The studies involving human participants were reviewed and approved by Kansas State University and University of Antioquia. Written informed consent to participate in this study was provided by the participants' legal guardian/next of kin.

## Author Contributions

BL-R, AL, HV, GV, EC, MF, and MC: study design and data analysis, writing, and reviewing. JM and AT-C: sample collection. KE-V, LR, and MB: mosquito rearing and salivary gland dissection. BL-R and JM: ELISA testing. All authors contributed to the article and approved the submitted version.

## Conflict of Interest

The authors declare that the research was conducted in the absence of any commercial or financial relationships that could be construed as a potential conflict of interest.

## Supplementary Material

Supplementary Table 1 Immunogenic peptides alignment between *An. darlingi* and other vectors of human disease.
