# Supplementary material for: Antibody Responses Against Anopheles darlingi Immunogenic Peptides in Plasmodium Infected Humans
Source: Front Cell Infect Microbiol. 2020 Aug 31;10:455. doi: 10.3389/fcimb.2020.00455 (PMC7488213; doi:10.3389/fcimb.2020.00455)
Supplement: Supplementary Table 1 — Immunogenic peptides alignment between An. darlingi and other vectors of human disease. [file Data_Sheet_1.doc]

Query ID: lcl|Query_32093

**AnDar_PeroX1**

Query 1 RGQCDSTSPYRTYDGRCNNLQN 22

ETN66035.1 19 ...................... 40 An. darlingi (100%)

XP_311449.4 657 ....S.....F..T...... 676 An. gambiae (85%)

AAD22196.1 24 .NA......L..T...... 42 An. albimanus (79%)

EAT48446.1 93 E..PNY...S......... 111 Ae. aegypti (73%)

XP_001849762.1 95 ...F..S...... 107 Cx. quinquefaciatus (84%)

Query ID: lcl|Query_90620

**AnDar_PeroX2**

Query 1 GQCDSTSPYRTYDGRCNNLQNP 22

ETN66035.1 20 ...................... 41 An. darlingi (100%)

XP_311449.4 657 ....S.....F..T....... 677 An. gambiae (85%)

AAD22196.1 24 .NA......L..T....... 43 An. albimanus (80%)

EAT48446.1 93 E..PNY...S......... 111 Ae. aegypti (76%)

XP_001849762.1 95 ...F..S....... 108 Cx. quinquefaciatus (85%)

Query ID: lcl|Query_56444

**AnDar_Apy1**

Query 1 GGHSHSFLFSPD-SDQPYNKQDT 22

ETN63669.1 249 ............-.......... 270 An. darlingi (100%)

XP_001651910.2 255 .A......Y...-.K.. 270 Ae. aegypti (81%)

XP_001869672.1 257 .A......Y.RE-.GK..D.G.. 278 Cx. quinquefasciatus (64%)

CAB40345.1 251 .........-.NA.SK.H.Q... 272 An. gambiae (69%)

Query ID: lcl|Query_8153

**AnDar_Apy1**

Query 1 HMN---DLHARFDETSNKSSKC----RSD 22

ETN63669.1 39 ...---................----... 60 An. darlingi (100%)

CAA76821.1 43 ...---......A...ER.... 61 An. gambiae (84%)

XP_001858188.1 31 .L.---.F....E..NL..NN.TVSE.. 55 Cx. quinquefaciatus (56%)

XP_001651910.2 44 .I.---......E..NM..NA. 62 Ae. aegypti (68%)

*(%) denotes peptide identity
